# Non-coding RNAs in gastric cancer immunotherapy: mechanisms and clinical implications

**DOI:** 10.3389/fimmu.2025.1688181

**Published:** 2025-10-28

**Authors:** Haotian Dong, Chengyuan Ye, Xuan Yu, Yongfu Shao

**Affiliations:** ^1^ Department of Gastroenterology, the First Affiliated Hospital of Ningbo University, Ningbo, China; ^2^ Health Science Center, Ningbo University, Ningbo, China

**Keywords:** ncRNAs, gastric cancer, tumor immunity, immunotherapy, molecular mechanism

## Abstract

Gastric cancer immunotherapy, recognized as the fourth primary treatment modality after surgery, radiotherapy, and chemotherapy, encompasses strategies such as immune checkpoint inhibitors and cellular immunotherapy and provides new avenues for cancer control. Recent studies have revealed that non-coding RNAs (ncRNAs), including miRNAs, lncRNAs, piRNAs, siRNAs, and circRNAs, drive the progression of gastric cancer primarily through three regulatory axes: epigenetic modification, transcriptional reprogramming, and tumor microenvironment remodeling. These processes are closely linked to tumor immunity and the efficacy of immunotherapy in gastric cancer (GC). Building on an overview of current immunotherapy regimens for GC, this review provides a comprehensive summary of the molecular mechanisms by which ncRNAs regulate immune cell infiltration, modulate immune checkpoints, and reshape the immunosuppressive microenvironment to influence immunotherapeutic outcomes. Furthermore, the potential translational applications of ncRNAs as prognostic biomarkers and therapeutic targets within the context of GC immunology are discussed. Collectively, these mechanistic insights and clinical perspectives offer a theoretical foundation for overcoming the limitations of current immunotherapy approaches and improving the long-term prognosis of patients with GC.

## 1 Introduction

According to the 2025 Global Cancer Statistics released by the International Agency for Research on Cancer (IARC) Global Cancer Observatory (GLOBOCAN), gastric cancer ranks fifth in both global cancer incidence and mortality (1). Notably, East Asian countries account for a disproportionately high incidence rate of 71.4%, a striking epidemiological disparity attributed to region-specific risk factors, including distinct dietary patterns, the prevalence of Helicobacter pylori infection, and population-specific genetic susceptibilities (2). Although the widespread adoption of endoscopic screening has improved early detection rates, the insidious onset and aggressive nature of gastric cancer result in most patients being diagnosed at advanced stages, contributing to a persistently poor 5-year survival rate of less than 30% (3). Advances in immuno-oncology and tumor microenvironment (TME) research have identified tumor immune escape mechanisms as critical therapeutic targets (4, 5). Cancer cells orchestrate immunosuppression by dysregulating T-cell effector functions and overexpressing immune checkpoint molecules while simultaneously evading immune surveillance through selective loss of highly immunogenic antigens (6). Emerging evidence positions immunotherapy as a promising treatment option for advanced gastric cancer. Although immune checkpoint inhibitors offer superior prognostic outcomes compared to conventional therapies by reactivating the host immune response against tumor cells, the heterogeneity of the TME contributes to therapeutic resistance, posing a significant clinical challenge (7, 8). This therapeutic paradox underscores the urgent need to elucidate the molecular determinants of immunotherapy responsiveness in gastric cancer.

Non-coding RNAs (ncRNAs) are a class of RNA molecules that are not translated into proteins and exhibit dynamic expression patterns in human cells. These include miRNA, lncRNA, circRNA, siRNA, and piRNA, whose abundance and regulatory functions profoundly influence cellular homeostasis (9, 10). Molecular biology studies have revealed that specific ncRNAs perform distinct biological roles through complex interactome networks (11), such as modulating mRNA translation via RNA-binding proteins (RBPs), regulating gene expression as competing endogenous RNAs (ceRNAs), and fine-tuning critical signaling pathways (12, 13). Recent foundational research has demonstrated that various non-coding RNA subtypes regulate gene expression in gastric cancer through epigenetic, transcriptional, and post-transcriptional mechanisms, thereby contributing to tumorigenesis and influencing tumor immunity (14, 15).

This review provides a comprehensive overview of the biological functions of ncRNAs and current immunotherapeutic strategies for gastric cancer. This review highlights specific ncRNAs that modulate downstream targets affecting the tumor immune response by regulating immune cell infiltration, altering immune checkpoint expression, and remodeling the immunosuppressive microenvironment. At the clinical level, ncRNAs hold potential as biomarkers for predicting immunotherapy outcomes and as therapeutic targets, offering precise strategies for intervention in gastric cancer treatment.

## 2 Overview of ncRNAs

### 2.1 Concept and types of ncRNAs

NcRNAs are ubiquitous in eukaryotic organisms and directly regulate cellular processes at the post-transcriptional level (16). Unlike messenger RNAs (mRNAs), which serve as carriers of genetic information, ncRNAs regulate various cellular functions through various mechanisms, including gene expression modulation, chromatin remodeling, RNA splicing, and translational control (17). Groundbreaking genomic studies have redefined conventional understanding: although only about 2% of the human genome encodes proteins, recent data from the ENCODE project reveal that over 76% of genomic DNA is actively transcribed into ncRNAs. These ncRNAs display tissue-specific expression patterns and are highly responsive to disease states (18). The advent of high-throughput sequencing technologies has further illuminated the crucial role of ncRNAs in tumorigenesis (19). For example, lncRNA H19, which is abnormally expressed in gastric cancer, influences the infiltration of cancer-associated fibroblasts (CAFs), macrophages, CD4+T cells, and CD8+T cells in the TME through the miR-378a-5p/SERPINH1 axis, thereby promoting the progression of gastric cancer (20). These findings underscore the strategic significance of ncRNAs in cancer immunotherapy and highlight their potential in the development of RNA interference-based targeted therapies.

**Figure 1:**
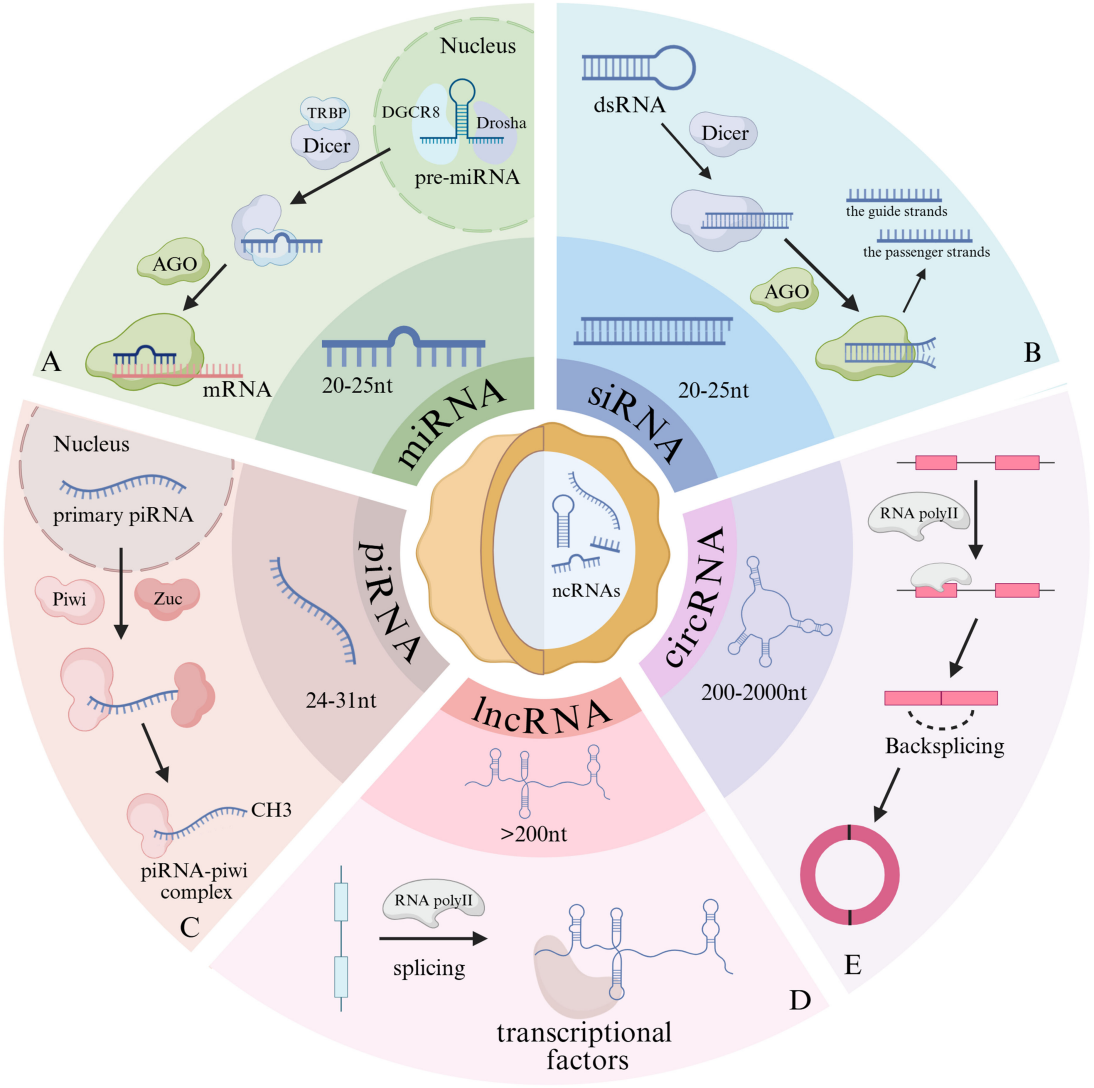
Classification of ncRNAs. **(A)** miRNAs are ncRNAs with a length of 20–25 nt, generated through Dicer, TRBP, and AGO-mediated cleavage of pre-miRNA. They function by binding to target mRNAs in a sequence-specific manner, resulting in either transcript degradation or translational repression. **(B)** siRNAs, approximately 20–25 nt in length, are produced by Dicer-mediated dsRNA cleavage and are composed of a guide strand and a passenger strand. **(C)** piRNAs, measuring 26-31nt, associate with PIWI proteins. Primary piRNAs undergo Zuc-dependent cleavage to generate mature piRNAs, which are subsequently methylated at the 3′-end. **(D)** LncRNAs are transcripts over 200 nt in length, typically synthesized by RNA polymerase II. **(E)** CircRNAs are covalently closed circular RNA molecules, generally 200-2000nt in length.

NcRNAs are broadly classified into small non-coding RNAs (sncRNAs), long non-coding RNAs (lncRNAs), and circular RNAs (circRNAs), based on their molecular length and functional characteristics (21). The sncRNA category primarily includes microRNAs (miRNAs), small interfering RNAs (siRNAs), and PIWI-interacting RNAs (piRNAs) (21). miRNAs, typically 20–25 nt in length, mediate translation inhibition or RNA degradation by partially binding to the 3 ‘-untranslated region (3 ‘-UTR) of target mRNAs, thereby regulating processes such as cell proliferation and apoptosis (22) (**Figure 1A**). In contrast, siRNAs induce gene silencing by forming perfect complementary base pairs with target mRNAs, resulting in mRNA degradation and the suppression of homologous gene expression (23) (**Figure 1B**). Leveraging this gene-silencing mechanism, siRNA-based technologies are now being incorporated into the development of immune checkpoint blockade therapies (24). piRNAs, 24–31 nucleotides in length, are germ cell-specific ncRNAs that associate exclusively with PIWI protein family members to form piRNA-PIWI complexes, which are essential for maintaining genomic stability and regulating translation (25) (**Figure 1C**). lncRNAs are defined as RNA transcripts longer than 200 nucleotides (26, 27) and are predominantly transcribed by RNA polymerase II (Pol II) (28) (**Figure 1D**). CircRNAs are mainly produced by RNA Pol II transcription. They characterized by their covalently closed loop structures, possess distinct regulatory functions by acting as molecular sponges that sequester miRNAs or by interacting with RNA-binding proteins (29, 30) (**Figure 1E**). Together, these ncRNA classes function as a complex “RNA regulatory code” with critical implications for advancing precision medicine.

### 2.2 The biological functions of ncRNAs

ncRNAs have emerged as critical regulators of various biological processes. Their functions extend beyond epigenetic modification, transcriptional, and post-transcriptional regulation to include complex roles in TME remodeling (31). Mechanistic studies have shown that distinct ncRNA subtypes drive gastric cancer progression through specific molecular interaction networks, profoundly impacting clinical outcomes and prognosis (32).

**Figure 2:**
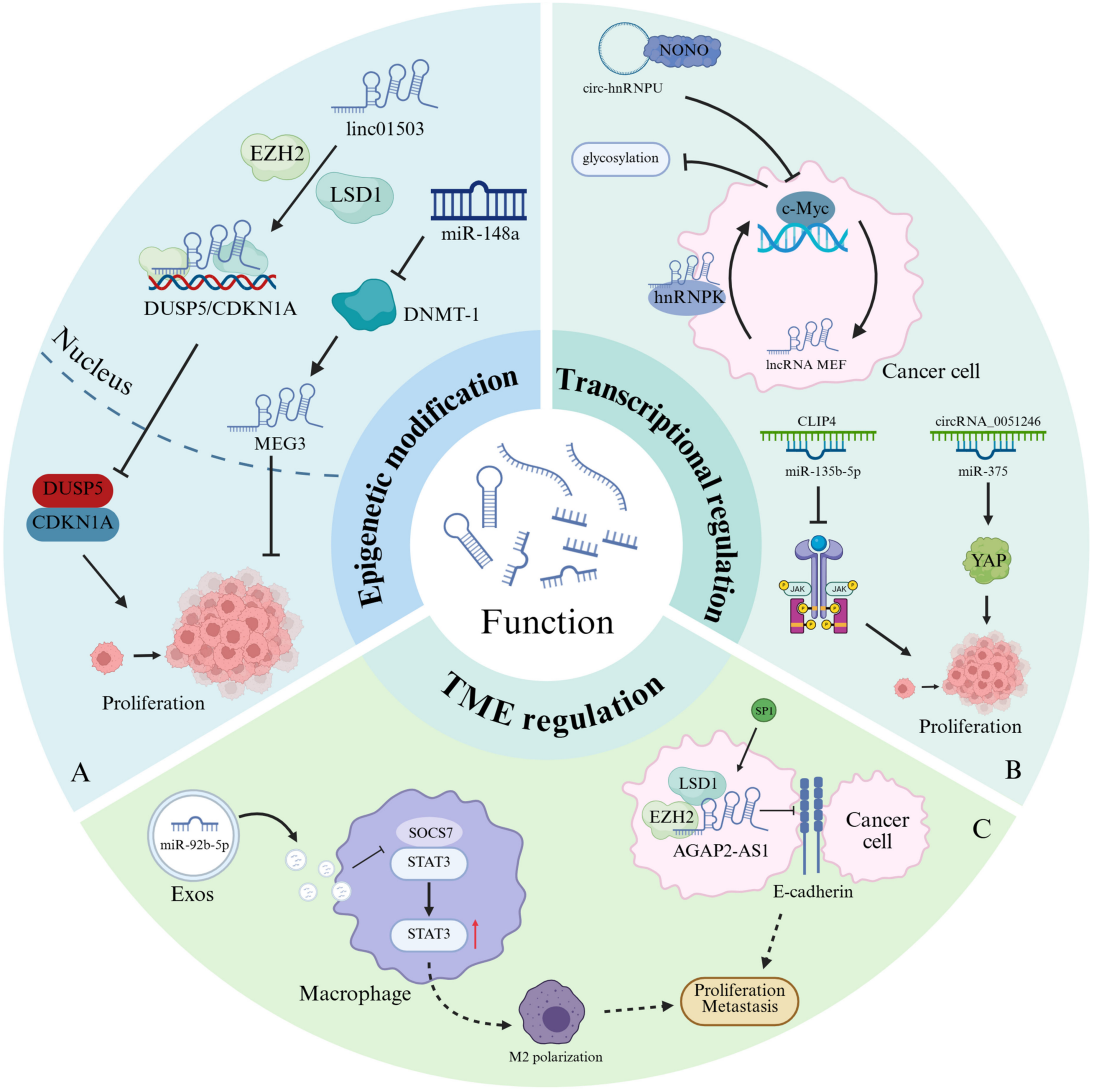
Biological functions of ncRNAs in gastric cancer. **(A)** Epigenetic regulation: LincRNA1503 recruits EZH2 and LSD1 to the promoter regions of DUSP5 and CDKN1A, inducing histone methylation that silences these tumor-suppressor genes and promotes the proliferation of gastric cancer cells. MiR-148a binds to DNMT1, reversing the hypermethylation of the MEG3 promoter and restoring the expression of this tumor-suppressive lncRNA, thereby inhibiting gastric carcinogenesis. **(B)** Post-transcriptional control: Circ-hnRNPU interacts with the NONO protein to suppress c-Myc transcription, thereby reducing the glycosylation of surface proteins in gastric cancer cells. LncRNA MEF forms a complex with hnRNPK, enhancing the translational efficiency of c-Myc mRNA and amplifying the oncogenic MEF-c-Myc feedback loop. MiR-135b-5p targets CLIP4, attenuating the JAK2/STAT3 signaling pathway and facilitating tumor proliferation. CircRNA_0051246 competitively binds miR-375, derepressing the proto-oncogene YAP and accelerating gastric cancer progression. **(C)** Tumor microenvironment modulation: Exosomal miR-92b-5p, internalized by TAMs, disrupts SOCS7-STAT3 binding, activating STAT3 signaling to drive M2 polarization and promote gastric cancer growth. SP1-activated lncRNA AGAP2-AS1 recruits LSD1 and EZH2 to repress E-cadherin transcription, impairing intercellular adhesion and facilitating tumor dissemination.

At the epigenetic regulation level, lncRNAs dynamically shape the epigenetic landscape by recruiting chromatin-modifying complexes (**Figure 2A**). For example, the oncogenic lncRNA linc01503 in gastric cancer interacts with zeste homolog 2 (EZH2) and lysine (K)-specific demethylase 1A (LSD1) enhancers, targeting the promoter regions of dual-specificity phosphatase 5 (DUSP5) and cyclin-dependent kinase inhibitor 1A (CDKN1A). This interaction induces histone H3 lysine 27 trimethylation (H3K27me3), leading to the epigenetic silencing of these tumor suppressor genes. Such epigenetic reprogramming inactivates cell cycle checkpoints, thereby enhancing the oncogenic potential of gastric cancer cells (32). In contrast, the tumor-suppressive lncRNA MEG3 exerts its function through a different epigenetic mechanism. miR-148 inhibits the translation of DNA methyltransferase 1 (DNMT1), thereby reversing hypermethylation at the MEG3 promoter. This reactivation restores MEG3 expression and suppresses gastric cancer progression (33).

Post-transcriptional regulation represents a central molecular mechanism by which ncRNAs drive oncogenesis through modulation of mRNA stability, translational efficiency, and protein function dynamics (34–36) (**Figure 2B**). For instance, circ-hnRNPU, which is downregulated in gastric cancer cells, binds to the NONO protein and competitively inhibits its interaction with the 3′-UTR of c-Myc mRNA. This dual mechanism suppresses c-Myc transcription and destabilizes its mRNA, thereby impeding abnormal glycosylation of surface proteins in gastric cancer cells (37). In colorectal cancer, the overexpression of lncRNA MEF enhances c-Myc mRNA translational efficiency by interacting with heterogeneous nuclear ribonucleoprotein K (hnRNPK), establishing the MEF-c-Myc regulatory axis as a driver of tumorigenesis (38). Similarly, the upregulation of miR-135b-5p in gastric cancer promotes tumor invasiveness by targeting CLIP4, thereby impairing the tumor-suppressive JAK2/STAT3 signaling pathway (39). The molecular sponge function of circRNAs is particularly prominent in post-transcriptional regulation. CircRNA_0051246, enriched with miR-375 binding sites, competitively sequesters miR-375, preventing it from silencing the proto-oncogene YAP1. This axis increases YAP protein levels, promoting self-renewal of cancer stem cells and accelerating gastric cancer progression (40).

In the context of TME remodeling, exosome-derived ncRNAs facilitate immune suppression via intercellular communication (**Figure 2C**). Gastric cancer cells secrete miR-92b-5p in exosomes, which are taken up by tumor-associated macrophages (TAMs). There, miR-92b-5p disrupts the SOCS7-STAT3 interaction, activating STAT3 signaling and inducing the M2 polarization of TAMs, which in turn promotes the proliferation of gastric cancer cells (41). Furthermore, the oncogenic lncRNA AGAP2-AS1, which is transcriptionally activated by SP1 in gastric cancer cells, forms a complex with LSD1 and EZH2 to repress E-cadherin mRNA expression. This repression reduces cell-cell adhesion and triggers epithelial-mesenchymal transition (EMT) (42).

## 3 Current advances in immunotherapeutic regimens for gastric cancer

### 3.1 Immune checkpoint inhibitors

**Figure 3:**
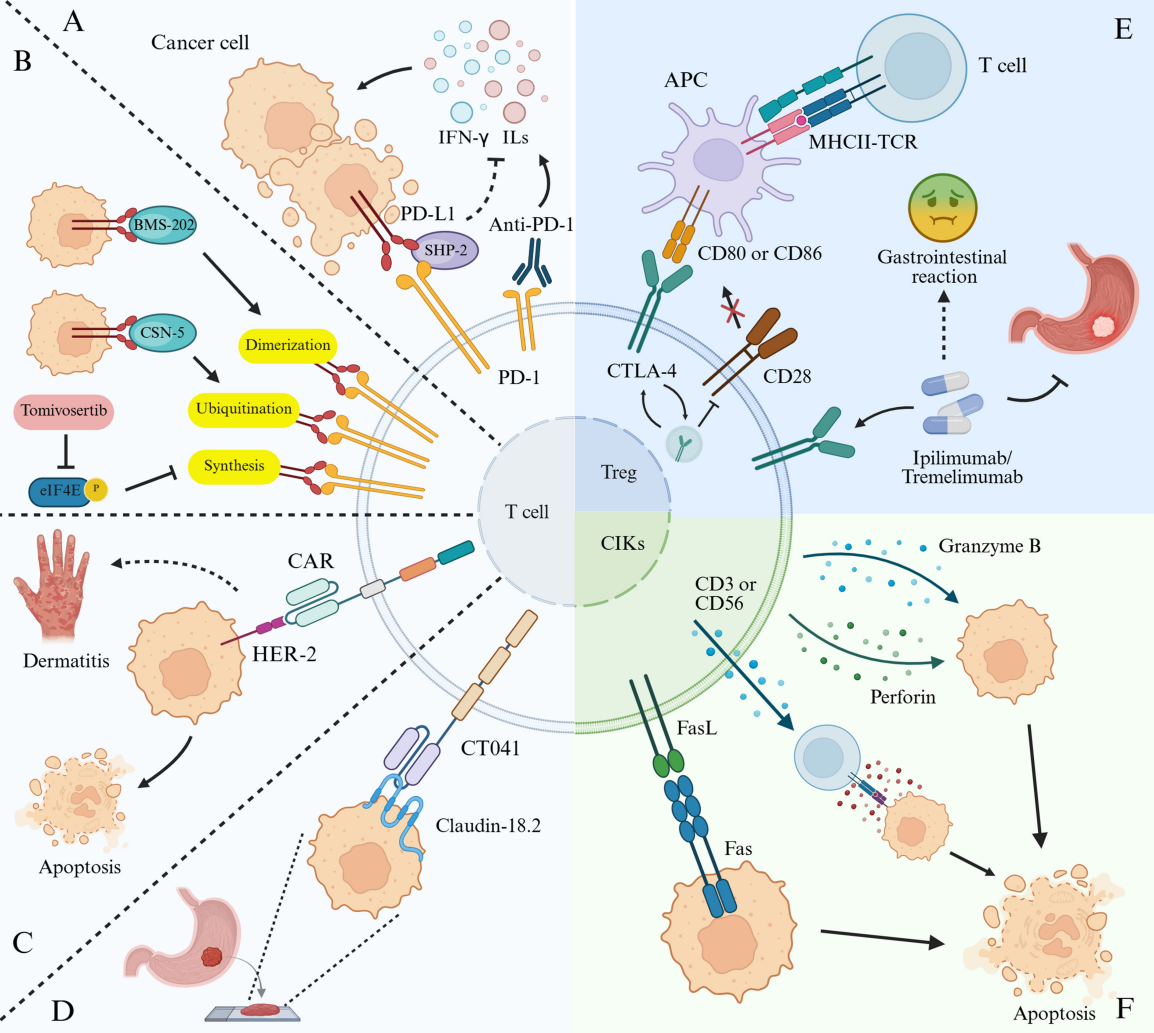
Molecular mechanisms of immunotherapies in gastric cancer. **(A)** PD-L1 regulation by cytokines: Tumor cell surface PD-L1 expression is upregulated by T cell-derived cytokines (e.g., IFN-γ, ILs) through SHP-2-mediated signaling. Anti-PD-1 antibodies block this axis by binding PD-1, thereby reducing PD-L1 expression and inhibiting tumor immune evasion. **(B)** Pharmacologic disruption of PD-L1 function: The agent targets PD-L1 through distinct mechanisms; for example, BMS-202 impairs dimerization, CSN5 inhibits ubiquitination, and tomivosertib suppresses synthesis. Collectively, these actions prevent the engagement of PD-L1/PD-1. **(C)** CAR-T therapy targeting HER2: CAR-T cells directed against HER2 epitopes effectively suppress gastric cancer progression, although treatment may cause mucositis. **(D)** CT041 targeting CLDN18.2: CT041 specifically targets the CLDN18.2 antigen, allowing for the selective elimination of tumor cells. **(E)** CTLA-4 pathway blockade: CTLA-4 on regulatory T cells (Tregs) binds CD80/CD86 on antigen-presenting cells (APCs), initiating endocytosis that depletes costimulatory ligands and inhibits CD28 signaling. Anti-CTLA-4 antibodies (ipilimumab and tremelimumab) block this interaction, suppressing tumor growth despite potential adverse effects such as nausea and vomiting. **(F)** CIK cells mediate tumor cell apoptosis via multiple mechanisms: the release of cytotoxic molecules such as perforin, granzyme B, CD3 and CD56, and the engagement of Fas ligand on CIK cells with Fas receptors on tumor cells.

While conventional therapeutic approaches offer limited survival benefits in gastric cancer, the persistently poor prognosis underscores the urgent need for more effective treatment strategies. The advent of ICI therapy has revolutionized the management of advanced-stage malignancies, demonstrating superior clinical efficacy in refractory and metastatic tumors compared with traditional treatments (43, 44). Mechanistically, immune checkpoints facilitate tumor immune evasion by engaging specific ligands that attenuate T-cell activity. ICIs disrupt this immunosuppressive pathway by targeting checkpoint molecules, thereby restoring T-cell-mediated antitumor responses (45) (**Figure 3A**). Currently, U.S. FDA-approved ICIs are classified into two major categories based on their molecular targets: programmed death-1/programmed death-ligand 1 (PD-1/PD-L1) inhibitors (e.g., nivolumab, pembrolizumab, and durvalumab) and cytotoxic T-lymphocyte-associated protein 4 (CTLA-4) inhibitors (e.g., ipilimumab and tremelimumab) (46). Recent clinical trials have rigorously evaluated the efficacy of ICIs in advanced gastric cancer. In the ATTRACTION-2 trial, Kang et al. demonstrated that nivolumab combined with chemotherapy significantly improved progression-free survival (PFS) and overall survival (OS) compared with chemotherapy alone (OS: 5.26 vs 4.14 months; HR = 0.63, p<0.0001) (47). Similarly, the KEYNOTE-859 trial led by Sun et al. showed that pembrolizumab plus chemotherapy resulted in a 1.4-month OS improvement over standard chemotherapy (median OS: 12.9 vs. 11.5 months; HR = 0.78, p<0.0001), establishing this combination as a new standard of cure (48). These paradigm-shifting outcomes have redefined the therapeutic landscape of advanced gastric cancer, advancing the field toward precision immuno-oncology.

Programmed death 1 (PD-1), an immunosuppressive receptor predominantly expressed on T-cells (49), binds to its principal ligand, programmed death ligand 1 (PD-L1), which is frequently overexpressed in malignant tumors—particularly in advanced gastric cancer associated with Epstein-Barr virus (EBV) (50, 51). Upon PD-1 binding to PD-L1, the phosphatase SHP-2 is recruited, resulting in the disruption of downstream T-cell receptor signaling. Consequently, T-cell activation is suppressed, thereby inhibiting the production of cytokines such as interferon-γ (IFN-γ) and interleukins (ILs). This pathway sustains immune tolerance and facilitates tumor immune escape (52, 53). Targeting the PD-1/PD-L1 axis has become a central focus in cancer immunotherapy, with monoclonal antibodies (mAbs) such as pembrolizumab and nivolumab receiving FDA approval since 2014 (54, 55). However, conventional mAb-based therapies face limitations, including inadequate tumor penetration (56) and systemic adverse effects such as gastrointestinal toxicity, endocrine dysregulation, and dermatologic reactions (57). These limitations have spurred interest in small-molecule inhibitors targeting the PD-1/PD-L1 pathway. BMS-202, a non-peptidic compound, disrupts PD-1/PD-L1 interaction by inducing PD-L1 dimerization (58) (**Figure 3B**). Complementing this extracellular targeting approach, eFT508 (Tomivosertib) acts at the translational level by inhibiting phosphorylation of eukaryotic initiation factor 4E (eIF4E) at Ser209, thereby reducing PD-L1 protein synthesis in gastric cancer organoids (59) (**Figure 3B**). Additionally, curcumin mediates post-translational PD-L1 regulation by promoting its ubiquitination via the COP9 signalosome subunit 5 (CSN5), which accelerates PD-L1 degradation (60) (**Figure 3B**).

Cytotoxic T-lymphocyte-associated protein 4 (CTLA-4), a ligand-independent immune checkpoint, plays a critical role in regulatory T cell (Treg)-mediated immunosuppression (61) (**Figure 3E**). Patients with gastric cancer exhibit significantly elevated Treg levels compared to healthy individuals, with Treg infiltration increasing across tumor stages, an indicator of poor prognosis. CTLA-4, expressed on Tregs, binds to CD80 and CD86 ligands on antigen-presenting cells (APCs), triggering the internalization of the CTLA-4 complex. This endocytosis depletes costimulatory ligands from the APC surfaces, inhibiting CD28-mediated T-cell activation and facilitating systemic immune suppression (62). Clinically approved anti-CTLA-4 monoclonal antibodies (mAbs), including ipilimumab and tremelimumab, have demonstrated varied efficacy profiles. In melanoma, ipilimumab significantly improves overall survival and progression-free survival compared with PD-1/PD-L1 inhibitors (63), although this benefit is associated with a 5% higher incidence of immune-related adverse events (64). In contrast, trials in gastrointestinal cancers have reported limited efficacy of tremelimumab in metastatic gastric and esophageal cancers, along with severe toxicities including nausea, vomiting, and rare cases of bowel perforations (65). These findings underscore the need for the development of next-generation CTLA-4 inhibitors that strike a balance between therapeutic effectiveness and improved safety.

### 3.2 Immunotherapy for cellular cancer

Tumor cells secrete immunosuppressive cytokines, such as interleukin-10 (IL-10), prostaglandin E2, and lymphocyte-activation gene 3 (LAG-3), thereby promoting immune evasion (66). Cellular immunotherapy is a transformative therapeutic approach in which autologous immune cells are genetically engineered to acquire tumor-specific recognition and cytotoxic capabilities (67). In gastric cancer, two primary cellular immunotherapeutic strategies have emerged as mainstays in clinical practice: adoptive cell therapy (ACT) and tumor vaccine-based approaches (68). These therapies activate tumor-specific cytotoxic T lymphocytes (CTLs) to lyse tumor cells and neutralize malignant cells by targeting surface antigens (69). The identification of multiple gastric cancer-associated tumor antigens has enabled the development of next-generation regimens for advanced disease, facilitating precision immunotherapies that enhance tumor immunogenicity while mitigating immune tolerance.

Adoptive cell therapy (ACT) involves isolating, modifying, and reinfusing a patient’s immune cells to directly kill tumor cells or amplify antitumor immune responses (70). Current ACT strategies employ various immune cell types, including tumor-infiltrating lymphocytes (TILs), cytokine-induced killer (CIK) cells, and chimeric antigen receptor (CAR) T cells (71, 72). TILs, which comprise heterogeneous populations of T cells, B cells, and natural killer (NK) cells, reflect the immune reactivity of the host to tumors (73). Clinical studies in advanced gastric cancer indicate that high TIL infiltration serves as an independent prognostic marker for overall survival, and is associated with prolonged OS (49, 74). CIK cells, characterized by dual CD3+CD56+ membrane expression and rapid ex vivo expansion, demonstrate potent tumoricidal activity (75, 76) (**Figure 3F**). In a phase II trial of metastatic gastric cancer, CIK cell infusion combined with FOLFOX4 chemotherapy significantly improved the objective response rates and quality-of-life scores (77). Similarly, intraperitoneal CIK administration with chemotherapy reduced the volume of malignant ascites and extended median OS in patients with gastric cancer with peritoneal carcinomatosis without increasing toxicity (78). As key mediators of tumor immunosurveillance, NK cells play a crucial role in suppressing gastric cancer progression and metastasis. Ishigami et al. conducted a retrospective analysis of 146 gastric cancer specimens and found that high NK cell infiltration was associated with lower TNM stage, reduced lymphovascular invasion, and improved five-year survival (79). Despite promising results, ACT in gastric cancer requires further optimization, including the development of standardized cell expansion protocols, optimization of dosing regimens, and strategies to mitigate cytokine release syndrome (CRS) in CAR-T therapy (80).

CAR-T therapy, a leading form of ACT, has been extensively used in refractory and recurrent solid tumors (81). This approach involves genetically modifying patients’ T cells to express chimeric antigen receptors (CARs) (82), enabling MHC-independent tumor recognition and cytotoxic activity against antigen-expressing cancer cells (83). Human epidermal growth factor receptor 2 (HER2), which is frequently overexpressed in gastric cancer, has been targeted in CAR-T trials, resulting in partial remission and an improved prognosis (**Figure 3C**). However, dermatitis and mucositis have been reported as adverse effects (84). Claudin 18.2 (CLDN18.2), a tight junction protein highly expressed in gastric adenocarcinomas and metastases (85), has emerged as another promising target. In preclinical models, CLDN18.2-specific CAR-T cells inhibited tumor growth with favorable safety profiles (86). Satricabtagene autoleucel (satri-cel, CT041), a first-in-class CLDN18.2-targeted CAR-T therapy, is currently undergoing global clinical trials (**Figure 3D**). In a phase I study, Qi et al. reported an objective response rate (ORR) of 57.1% and a disease control rate (DCR) of 75%, with a six-month OS rate of 81.2% in gastric cancer patients (87). By comparison, third-line treatments, such as nivolumab, pembrolizumab, trifluridine/tipiracil, and apatinib, yield ORRs of 11.2%, 13.3%, 4%, and 1.7%, respectively, with median OS below six months (47, 88–90). These results position CT041 as a potentially transformative option in third-line gastric cancer therapy, showing superior efficacy to conventional regimens in refractory advanced or metastatic disease (91). Mechanistically, Samer et al. demonstrated that anti-mesothelin hYP218 CAR-T cells persisted within the tumor microenvironment and retained cytotoxicity, with the co-administration of pembrolizumab enhancing antitumor activity in advanced gastric cancer (92). Beyond HER2 and CLDN18.2, emerging CAR-T targets include mesothelin (MSLN), carcinoembryonic antigen (CEA), and epithelial cell adhesion molecule (93).

Despite substantial advances, cellular immunotherapy in gastric cancer still faces challenges such as high treatment costs and unresolved safety concerns. Current research focuses on next-generation CAR-T platforms with enhanced tumor specificity and multimodal regimens combining cellular therapies with complementary treatments. These strategies aim to enhance the efficacy, safety, and cost-effectiveness of gastric cancer management.

## 4 The molecular mechanism of ncRNAs in gastric cancer immunotherapy

### 4.1 Regulation of immune cell infiltration

**Figure 4:**
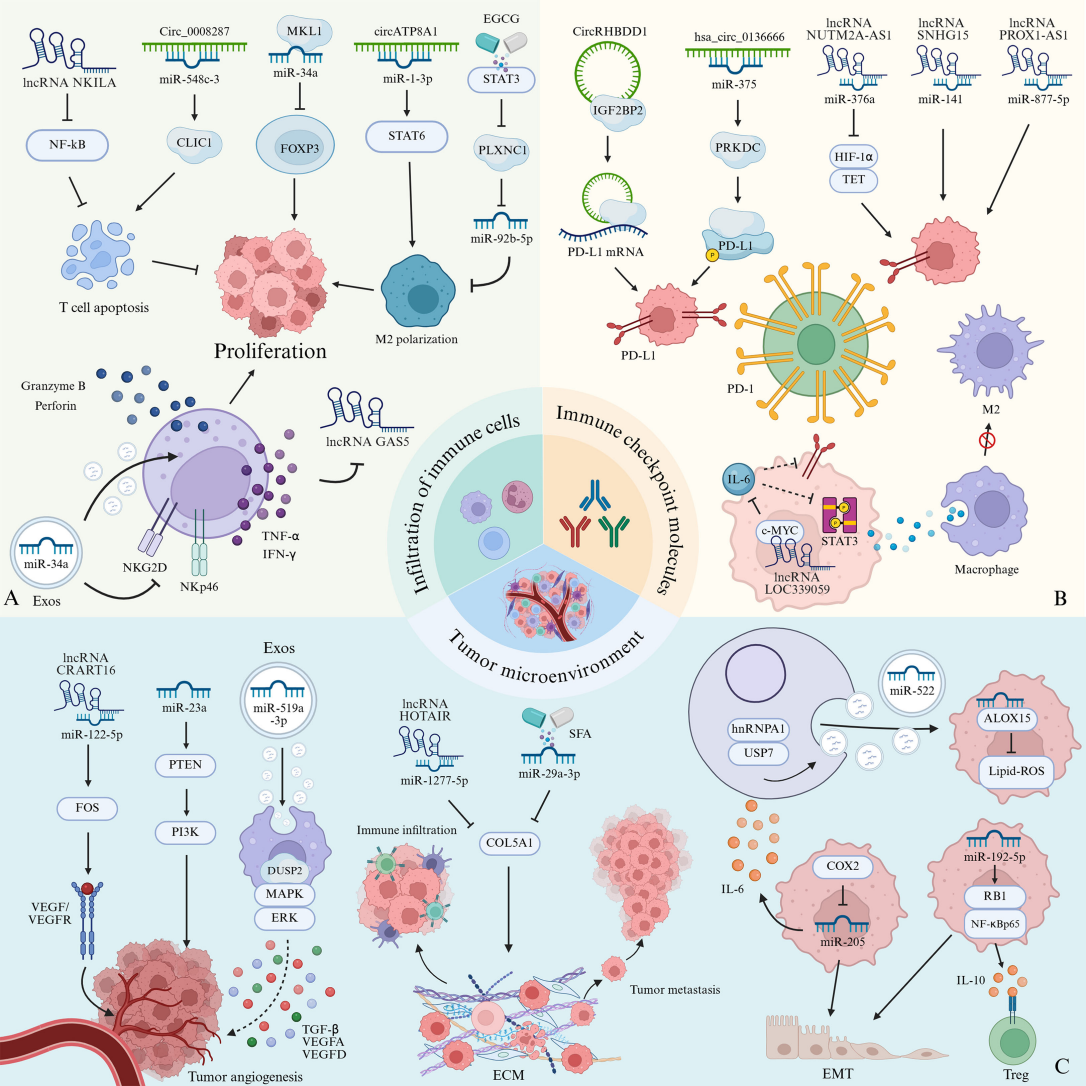
Molecular mechanisms of ncRNAs in gastric cancer immunotherapy. **(A)** Immune cell infiltration modulation: LncRNA NKILA suppresses T cell apoptosis and tumor proliferation by inhibiting the NF-κB pathway. Circ_0008287 binds to miR-548c-3, relieving its inhibition of CLIC1 and thereby promoting immune cell apoptosis and accelerating tumor progression. MiR-34a interacts with MKL1 to repress FOXP3, facilitating tumor cell proliferation. CircATP8A1 enhances STAT6-mediated M2 macrophage polarization by sponging miR-1-3p. Conversely, EGCG inhibits STAT3-dependent PLXNC1 expression, reducing miR-92b-5p levels and suppressing M2 polarization. Exosomal miR-552-5p represses NKG2D and NKp46 transcription, thereby impairing NK cell cytokine production (IFN-γ, TNF-α, perforin, and granzyme B). IL-2-stimulated NK cells downregulate GAS5 lncRNA expression. **(B)** Immune checkpoint regulation: CircRHBDD1 stabilizes PD-L1 mRNA expression via IGF2BP2. Hsa_circ_0136666 relieves miR-375-mediated suppression of PRKDC by enhancing PD-L1 phosphorylation. Collectively, these mechanisms elevate PD-L1 expression, promoting immune evasion. LncRNAs (NUTM2A-AS1/miR-376a, SNHG15/miR-141, and PROX1-AS1/miR-877-5p) upregulate PD-L1 to accelerate the progression of gastric cancer. In contrast, lncRNA LOC339059 inhibits c-Myc-dependent IL-6 transcription, thereby suppressing STAT3 phosphorylation and PD-L1 activation, ultimately impairing macrophage polarization and the activation of gastric cancer cells. **(C)** Tumor microenvironment reprogramming: In tumor angiogenesis, the lncRNA CRART16/miR-122-5p axis activates FOS-mediated VEGF/VEGFR2 signaling. MiR-23a promotes PI3K/Akt activation by targeting PTEN. Exosome-transferred miR-519a-3p induces DUSP2/MAPK/ERK-mediated M2 polarization, triggering the secretion of pro-angiogenic cytokines (TGF-β, VEGFA, VEGFD). In extracellular matrix remodeling, lncRNA HOTAIR and miR-29a-3p modulate COL5A1 expression, with the overexpression of HOTAIR promoting collagen deposition and ECM stiffening, while miR-29a-3p counteracts this effect. ECM stiffening impairs immune cell infiltration and facilitates tumor proliferation. In addition, USP7 deubiquitinates hnRNPA1 in CAFs, enabling the transfer of exosomal miR-522 to tumor cells, where it inhibits ALOX15, reduces lipid-ROS production, and suppresses ferroptosis. COX-2/HIF-1α-activated miRNA-205 induces IL-6 secretion and promotes EMT progression. MiR-192-5p activates RB1/NF-κBp65 signaling, thereby enhancing IL-10 secretion and regulating Treg differentiation and EMT.

NcRNAs act as central regulators of the gastric cancer tumor microenvironment, orchestrating immune cell infiltration and antitumor immune responses through epigenetic reprogramming, modulation of signaling pathways, and RNA-RNA/protein interaction networks (**Table 1**). These molecular regulators influence the recruitment, activation, and functional polarization of cytotoxic T lymphocytes (CTLs), regulatory T cells (Tregs), tumor-associated macrophages (TAMs), and myeloid-derived suppressor cells (MDSCs) (94). ncRNAs function as molecular hubs to maintain the balance among immune cell subpopulations (**Figure 4A**). For example, by suppressing NF-κB signaling, NKILA reduces the susceptibility of T cells to activation-induced cell death (AICD), thereby enhancing their antitumor capacity. However, clinical data indicate that gastric cancer patients with high NKILA expression in tumor-specific CTLs have shorter median overall survival than those with low expression, suggesting that lncRNA-based therapies may exert tumor microenvironment-specific effects and require further profiling for precision application (95). Conversely, circ_0008287, which is overexpressed in gastric tumors, promotes immune evasion by sponging miR-548c-3p to derepress chloride intracellular channel 1 (CLIC1). This axis impairs CD8+ T-cell function, reduces IFN-γ secretion, and accelerates tumor progression (96). FOXP3 is significantly overexpressed in gastric cancer tissues compared with adjacent normal mucosa (97). Tumor-associated FOXP3 competitively binds NF-κB p65, reducing its transcriptional activation of the tumor suppressor p21 and promoting cancer cell proliferation (98). The tumor-suppressive miR-34a, which is transcriptionally regulated by megakaryocytic leukemia 1 (MKL1), directly targets FOXP3 mRNA, thereby limiting Treg differentiation and its tumor-promoting effects (99). Similarly, miR-133a-3p binds to FOXP3, suppressing its expression and stimulating autophagy in gastric cancer cells, thereby contributing to the stability of the tumor microenvironment (100). miR-128-3p has been identified as a master immunoregulator that controls IL-16-dependent recruitment of CD4+CD25+FOXP3+ Tregs, thereby dampening antitumor immunity (101). These findings suggest that miRNA-mediated regulation of TIL subsets represents a potential direction for the development of tumor immunotherapy. Compared to adjacent tissues, gastric cancer lesions contain significantly more FOXP3+ Tregs. The cyclooxygenase-2 (COX-2) released by these cells induces the expression of prostaglandin E2, which suppresses the activity of effector T cells, providing an additional mechanism for immune evasion (102). Therefore, COX-2 inhibitors may reduce COX-2 expression and enhance effector T-cell function, offering another avenue for gastric cancer immunotherapy.

**Table 1:** Molecular mechanisms of ncRNAs in gastric cancer immunotherapy.

Molecular mechanism	ncRNA	Specific mechanism	Regulating targets	Function	Ref
Regulation of immune cell infiltration	NKILA lncRNA	Sensitize T cells to activation-induced cell death	NF-κB	up	(95)
	circ_0008287	Impair the function of CD8+T cell	CLIC1	up	(96)
	miR-34a	Limit Treg differentiation	FOXP3	down	(99)
	miR-133a-3p	Stimulate autophagy in GC cells	FOXP4	up	(100)
	circATP8A1	Promote M2 polarization	STAT6	up	(103)
	miR-92b-5p	Inhibit M2 polarization	STAT3/PLXNC1	down	(41)
	lncRNA GAS5	Inhibit NK cell cytotoxicity	IFN-γ/TNF-α	down	(104)
Regulation expression of immune checkpoint	circRHBDD1	Increase PD-L1 expression	IGF2BP2	up	(107)
	miR-375	Inhibit PD-L1 transcription	JAK2/STAT3	down	(108)
	hsa_circ_0136666	Phosphorylation of PD-L1	PRKDC	up	(109)
	lncRNA NUTM2A-AS1	Increase PD-L1 expression	TET1/HIF-1α	up	(111)
	LOC339059	Inhibit IL-6	c-Myc	down	(117)
Regulate tumor microenvironment	CRART16	Activate the VEGF/VEGFR2 axis	FOS	up	(120)
	miR-23a	Activate the PI3K/Akt pathway	PTEN	up	(121)
	HOTAIR	Promote ECM stiffening	COL5A1	up	(123)
	miR-205	Regulate EMT	IL-6	down	(128)
	miR-192-5p	Regulate EMT	RB1/NF-κBp65	up	(129)

Within the immunosuppressive tumor microenvironment, M2-polarized macrophages are critical mediators of immune evasion, with their polarization regulated by ncRNAs via exosome-mediated communication. CircATP8A1, enriched in gastric cancer-derived exosomes, sponges miR-1-3p, depressing STAT6 expression, which drives M2 polarization and accelerates tumor progression (103). Additionally, the natural polyphenol epigallocatechin gallate (EGCG) exerts dual immunomodulatory effects by transcriptionally repressing STAT3, thereby downregulating PLXNC1 expression and reducing exosomal miR-92b-5p levels in gastric cancer cells. In clinical specimens, the STAT3/PLXNC1 axis-regulated miR-92b-5p is strongly correlated with M2 macrophage infiltration (41). These findings establish a mechanistic basis for targeting the STAT3-PLXNC1-miR-92b-5p axis, with EGCG proposed as a potential synergistic partner for immune checkpoint inhibitors to overcome immunotherapy resistance in gastric cancer.

NK cells also play a pivotal role in antitumor immunity. Inflammatory cytokines, such as IL-2, enhance the secretion of TNF-α and IFN-γ by NK cells but downregulate the tumor-suppressive lncRNA GAS5. Luciferase assays confirm direct binding of GAS5 to miR-18a, and GAS5 depletion increases miR-18a expression, thereby reducing NK cell cytotoxicity. Restoring GAS5 levels significantly increases NK cell infiltration in gastric cancer tissues, supporting its potential in NK cell-based immunotherapy (104). Tang et al. described a dual inhibitory mechanism in which exosomal miR-552-5p suppresses the transcription of NK cell activation receptors NKG2D and NKp46, while decreasing the secretion of cytotoxic molecules IFN-γ and TNF-α. Simultaneously, miR-552-5p upregulates PD-L1 expression, thereby activating the PD-1/PD-L1 checkpoint and reducing the secretion of perforin and granzyme B. Notably, the PD-L1 inhibitor durvalumab can counteract the immunosuppressive effects of miR-522-5p, thereby restoring NK cell cytotoxicity. *In vivo* experiments in nude mice confirmed a significant reduction in tumor volume following treatment with an anti-PD-L1 antibody (105, 106). These results indicate that ncRNAs can induce NK cell exhaustion through a multi-level receptor-ligand-effector regulatory network, offering novel targets for immune checkpoint inhibitors in gastric cancer.

Based on these mechanistic insights, therapeutic strategies that silence oncogenic ncRNAs can restore CTL function, while restoring tumor-suppressive ncRNAs can counteract Treg-mediated immunosuppression. Together, these approaches can remodel the tumor microenvironment into a pro-immunogenic state. The integration of ncRNA modulation with current immunotherapies holds promise for improving response rates and advancing precision immuno-oncology.

### 4.2 Regulation of the expression of immune checkpoint molecule

The dysregulated expression of immune checkpoint molecules, particularly PD-1/PD-L1 and CTLA-4, represents a key mechanism of immune evasion in gastric cancer (**Table 1**). NcRNAs have emerged as critical modulators of this process through epigenetic reprogramming and post-transcriptional control (**Figure 4B**). CircRHBDD1, which is markedly upregulated in gastric cancer tissues, enhances PD-L1 mRNA stability by directly interacting with the RNA-binding protein IGF2BP2 in an m6A-dependent manner. This interaction increases PD-L1 protein levels and reduces CD8+ T-cell infiltration, thereby promoting tumor immune escape. Targeted delivery of circRHBDD1-specific siRNA via PLGA-PEG nanoparticles significantly suppressed tumor growth, enhanced the efficacy of anti-PD-L1 therapy, and reduced severe adverse effects in murine models (107). Similarly, miR-375, which is downregulated in gastric cancer, is inversely correlated with PD-L1 expression. Mechanistic analyses indicate that miR-375 inhibits PD-L1 transcription by suppressing JAK2/STAT3 signaling, as evidenced by reduced phosphorylated STAT3 levels following miR-375 overexpression (108). These findings provide a strong rationale for the development of ncRNA-targeted nanotherapeutics along with immune checkpoint blockade.

Emerging evidence shows that ncRNAs regulate immune checkpoints through multiple molecular pathways in gastric cancer. Hsa_circ_0136666, specifically overexpressed in gastric cancer, acts as a molecular sponge for miR-375. This interaction prevents the miR-375-mediated repression of the catalytic subunit of DNA-dependent protein kinase (PRKDC), triggering PRKDC-dependent phosphorylation of PD-L1 and thereby stabilizing PD-L1, which impairs CD8+ T-cell function. Preclinical studies using lipid nanoparticle (LNP)-encapsulated siRNA against hsa_circ_0136666, combined with anti-PD-L1 therapy, demonstrated synergistic tumor suppression, offering a potential strategy to overcome resistance to immune checkpoint inhibitors (109). In another pathway, lncRNA linc01094 dually regulates PD-L1 and PD-L2 expression by sponging miR-17-5p. Elevated PD-L2 expression in linc01094-high tumors induces M2 macrophage polarization and CD8+ T-cell exhaustion, creating an immunosuppressive niche (110). Similarly, lncRNA NUTM2A-AS1 binds to miR-376a, activating the TET1/HIF-1α axis and increasing PD-L1 expression, which confers cisplatin resistance. Combined treatment with cisplatin and anti-PD-L1 produced superior responses in advanced gastric cancer (111). Conversely, lncRNA SNHG15 promotes immune evasion by sponging miR-141, thereby derepressing PD-L1 translation in cancer stem cells, which correlates with diminished anti-PD-L1 efficacy (112). Similarly, PROX1-AS1 sustains PD-L1 expression by sequestering miR-877-5p, thereby driving tumor progression (113). Collectively, these studies identified PD-L1 as a central hub linking ncRNA networks to immune evasion and therapy resistance. A tumor-suppressive miRNA network can also foster an immunopermissive tumor microenvironment through multi-checkpoint regulation. miR-152, miR-93, and miR-545-5p directly target the 3′-UTRs of PD-L1, TIM-3, and B7-H4, respectively, downregulating their expression in gastric adenocarcinoma cells. This coordinated regulation establishes a multi-target inhibitory framework, suggesting new opportunities for combinatorial immunotherapy to overcome resistance to checkpoint blockade (114–116).

Notably, some ncRNAs demonstrate cross-dimensional regulatory capacity, modulating immune checkpoints and influencing the behavior of immune cells. LncRNA LOC339059 directly binds to c-Myc protein, inhibiting its transcriptional activation of IL-6, thereby preventing STAT3 phosphorylation and downstream PD-L1 expression. Elevated IL-6 expression correlates positively with the M2 macrophage markers CD206 and CD204; thus, LOC339059 overexpression suppresses M2 macrophage polarization through the IL-6/STAT3 axis (117). Conversely, exosomal miR-16-5p from M1 macrophages exerts a dual effect: it suppresses PD-L1 expression to activate T-cell-mediated tumor immunity and promotes repolarization of macrophages toward the M1 phenotype, thereby remodeling the tumor-immune microenvironment (118). These findings reveal that individual ncRNAs can concurrently regulate immune checkpoints and immune cell dynamics, adding complexity to ncRNA-mediated checkpoint networks. Targeting checkpoint-associated ncRNAs may provide novel strategies to overcome resistance to immune checkpoint inhibitors in gastric cancer, thereby supporting the development of stage-specific, precision immunotherapies.

### 4.3 Participation in the remodeling of the tumor microenvironment

The tumor microenvironment of gastric cancer is a dynamic ecosystem composed of stromal cells, extracellular matrix (ECM), and immunosuppressive mediators (**Table 1**). NcRNAs orchestrate TME remodeling through diverse mechanisms, including regulation of angiogenesis, ECM stiffening, and intercellular communication, exerting a pervasive influence throughout gastric cancer initiation and progression (**Figure 4C**).

Tumor angiogenesis, the formation of new blood vessels to supply nutrients and oxygen to malignant cells, is critically regulated by vascular endothelial growth factor-A (VEGF-A) (119). Mechanistically, lncRNA CRART16 acts as a molecular sponge for miR-122-5p, thereby relieving its inhibition of the target gene FOS and activating the VEGF/VEGFR2 axis, which promotes angiogenesis in gastric cancer (120). In parallel, exosomal miR-23a, markedly upregulated in gastric cancer, enhances endothelial cell proliferation and vascularization by targeting PTEN to activate the PI3K/Akt pathway (121). Notably, exosomes from gastric cancer cells with high hepatic metastatic potential are enriched in miR-519a-3p. These vesicles transfer miR-519a-3p to macrophages, thereby driving M2 polarization via the DUSP2/MAPK/ERK axis. Polarized macrophages secrete pro-angiogenic cytokines—including transforming growth factor-beta (TGF-β), VEGFA, and VEGFD—thereby increasing vascular permeability and neovascularization and establishing a nutrient-rich pre-metastatic niche that facilitates hepatic colonization. Thus, miR-519a-3p represents a promising biomarker for predicting hepatic metastasis, and its therapeutic inhibition may disrupt pre-metastatic niche formation (122).

Beyond angiogenesis, ncRNAs play a critical role in regulating extracellular matrix remodeling in gastric cancer. The long non-coding RNA HOTAIR, overexpressed in advanced tumors, promotes ECM stiffening by sponging miR-1277-5p, which derepresses the expression of collagen type V α1 chain (COL5A1), leading to excessive collagen deposition. This stiffened ECM enhances immune cell infiltration and accelerates metastasis in preclinical models (123). Conversely, miR-29a-3p counteracts this process by directly targeting COL5A1, thereby inactivating the Wnt/β-catenin pathway. Sulforaphane (SFA), a dietary isothiocyanate, further amplifies miR-29a-3p activity by promoting precursor miRNA transport to the cytoplasm, as confirmed by RNA immunoprecipitation (124). These findings support a dual-therapy strategy combining miR-29a-3p-loaded nanoparticles with ECM-degrading enzymes (e.g., collagenase) to overcome physical barriers to immunotherapy.

Intercellular communication in gastric cancer plays a crucial role in remodeling the tumor microenvironment. Recent studies have highlighted exosome-mediated ncRNA transfer between tumor cells and cancer-associated fibroblasts (CAFs) mediated (125). For example, CAF-secreted miR-522 binds to 15-lipoxygenase (ALOX15) mRNA, inhibiting lipid peroxidation and reducing ferroptosis in gastric cancer cells. Ubiquitin-specific protease 7 (USP7) enhances miR-522 secretion by deubiquitinating and stabilizing heterogeneous nuclear ribonucleoprotein A1 (hnRNPA1). Interestingly, cisplatin and paclitaxel can elevate ferroptosis levels in gastric cancer cells via the USP7/hnRNPA1/miR-522/ALOX15 axis, thereby enhancing chemosensitivity (126). Similarly, the upregulation of miRNA-106b in CAFs promotes tumor invasion and metastasis by targeting PTEN (127). CAF-derived ncRNAs also regulate EMT. miRNA-205, which is activated by the COX-2/HIF-1α axis, stimulates IL-6 secretion, which promotes CAF-induced EMT and contributes to fibrotic remodeling and immune exclusion within the tumor microenvironment (128). Elevated serum miR-192-5p levels correlate with advanced tumor stage and immunosuppressive phenotype. This miRNA enhances IL-10 secretion in gastric cancer cells by targeting the RB1/NF-κBp65 pathway, thereby facilitating Treg differentiation, regulating EMT, and remodeling the tumor immune microenvironment (129). These multifaceted regulatory roles highlight ncRNAs as promising therapeutic targets for disrupting the pro-tumor microenvironment. Integrating ncRNA modulation with stroma-targeted agents, such as angiogenesis inhibitors or ECM remodeling compounds, may enhance the efficacy of immunotherapy through reprogramming of the tumor microenvironment.

## 5 Clinical potential of ncRNAs in gastric cancer immunotherapy

### 5.1 Predictive biomarkers of immunotherapy prognosis

Early diagnosis is a critical determinant of clinical gastric cancer outcomes. Dysregulated ncRNAs in tumor tissues and circulation are closely associated with the remodeling of the immune microenvironment and therapeutic responses, making them promising biomarkers for early detection and prognostic stratification. Common detection methods involve the extraction of RNA from sources such as plasma and tissue samples, followed by reverse transcription and quantitative quantitative reverse transcription polymerase chain reaction (qRT-PCR) to verify the presence of differentially expressed targets. Deng et al. developed an immune-related prognostic model for gastric cancer based on competing endogenous RNA (ceRNA) network analysis, identifying three hub RNAs—lncRNA PVT1, miRNA hsa-miR-130a-3p, and mRNA RECK—through LASSO regression. The resulting risk score, lassoScore, was significantly higher in patients with gastric cancer than in healthy controls and correlated with poorer clinical outcomes. Mechanistic analysis linked high lassoScore to increased infiltration of resting memory CD4+ T cells, M2 macrophages, and resting mast cells. Moreover, RECK expression was associated not only with immune cells, such as CD8 T cells, mast cells, and NK cells, but also with immune checkpoints, including CD244, CD160, and PDCD1 (130). This model provides both molecular biomarkers and a theoretical framework for gastric cancer immunophenotyping and personalized therapy. Similarly, Xu et al. analyzed miRNA-immune gene networks in 389 patients with gastric cancer from The Cancer Genome Atlas (TCGA), identifying nine immune-related miRNAs with prognostic value (131). Multivariate Cox regression and ROC curve analyses classified six miRNAs (miR-125b-5p, miR-99a-3p, miR-145-3p, miR-328-3p, miR-133a-5p, and miR-1292-5p) as tumor suppressors and three (miR-675-3p, miR-92b-5p, and miR-942-3p) as oncogenic drivers. Kaplan-Meier analysis stratified patients into high- and low-risk groups, with the high-risk cohort showing significantly reduced survival. Immune profiling revealed distinct infiltration patterns of CD4+ T cells, macrophages, and dendritic cells, thereby validating these miRNAs as prognostic indicators. Prognostic models based on DNA damage repair-related lncRNAs or 11 m6A-modified lncRNAs have shown that patients with low-risk gastric cancer exhibit lower immune infiltration but improved responsiveness to immune checkpoint inhibitors and reduced therapeutic resistance (132, 133). These findings highlight the value of lncRNA-based models in predicting prognosis, immunotherapy sensitivity, and resistance mechanisms.

Liquid biopsy-based ncRNAs profiling offers a non-invasive platform for the real-time monitoring of gastric cancer progression and therapeutic response. Common liquid biopsy techniques involve the analysis of biomarkers in biofluids such as blood, urine, and saliva. Notably, neutrophil-derived miRNAs (miR-223-3p/miR-425-5p) demonstrated superior diagnostic performance (sensitivity: 77.05%; specificity: 77.51%) compared with conventional biomarkers and outperformed existing biomarkers in predicting metastatic potential and immunotherapy responsiveness (134). Exosomal circRNAs also display exceptional stability and can serve as dynamic biomarkers reflecting the status of the immune microenvironment in real-time (135). For instance, hsa_circ_0072309 modulates tumor immune infiltration via activation of the PI3K-AKT/Ras pathway (136), whereas circMAN1A2 (hsa_circ_0000118) influences gastric cancer progression by inhibiting CTL secretion of cytokines such as TNF-α and IFN-γ (137). Similarly, lncRNAs released into the extracellular environment can be detected in blood or other liquid biopsies (138). Ding et al. evaluated eight immune-related lncRNAs in a prognostic risk model and identified lncRNA RP11-617F23.1 as a key predictor of immune infiltration patterns and clinical outcomes in gastric cancer (139).

### 5.2 Targets of immunotherapy for gastric cancer

Beyond their established role as prognostic biomarkers, numerous ncRNAs hold substantial therapeutic potential as direct molecular targets in gastric cancer. This potential arises from their differential expression between malignant and adjacent normal tissues, coupled with their critical regulatory roles in tumorigenesis and metastasis. Targeting ncRNAs to reprogram the immunosuppressive tumor microenvironment is emerging as a promising strategy to enhance the efficacy of immunotherapy. For instance, Zheng et al. demonstrated that modulating miR-21 expression in post-gastrectomy patients alleviates surgery-induced Th17/Treg imbalance via PD-L1 axis regulation, suggesting that miR-21 inhibition is a viable therapeutic approach for mitigating postoperative immune dysfunction in advanced gastric cancer (140). Similarly, miR-105-5p, epigenetically regulated through GABRA3-mediated promoter hypomethylation, functions as a tumor suppressor by directly binding to the PD-L1 3’UTR and enhancing CD8+ T-cell cytotoxicity (141). These findings position miR-105-5p as a compelling candidate for immunotherapeutic intervention in gastric cancer.

The covalently closed circular structure and intrinsic RNase resistance of circRNAs confer unique advantages for therapeutic application in gastric cancer. Recent studies on ncRNA-mediated immunomodulation have identified critical circRNA-miRNA regulatory axes (e.g., hsa_circ_0061695 and hsa_circ_0091994) that reprogram the TME via immune cell modulation. Dysregulation of these axes promotes malignant progression by subverting immune surveillance and reshaping the tumor microenvironment. ICI therapy or cell-targeted approaches may counter these effects and reduce drug resistance (142). Furthermore, circRHBDD1 promotes immune evasion by enhancing IGF2BP2-mediated stabilization of PD-L1 transcripts, thereby reducing CD8+ T-cell infiltration in gastric cancer. To disrupt this axis, researchers have developed tumor-targeted nanoparticles based on poly (lactide-co-glycolic acid)-polyethylene glycol (PLGA-PEG) for the precise delivery of circRHBDD1-specific siRNA. This system not only induces tumor-specific immune memory but also acts synergistically with PD-1 inhibitors, demonstrating superior tumor-targeting efficiency (107). These advances underscore the need for advanced delivery systems, such as lipid nanoparticles or siRNA conjugates, to improve the stability and *in vivo* performance of ncRNA-based therapeutics (143).

### 5.3 Convergent ncRNA-immunotherapy strategies in gastric cancer

The therapeutic integration of ncRNA modulators with established immunotherapies represents a promising strategy for counteracting immune evasion and achieving durable clinical responses in gastric cancer. For example, combinatorial targeting of hsa_circ_0076092 with PD-1 blockade synergistically disrupts the miR-744-5p/SLC7A5 immunosuppressive axis, resulting in greater tumor growth inhibition than monotherapy (144). However, this strategy has not yet entered clinical trials. Similarly, SHP2 phosphatase activates the ROS/JNK/NFAT4 signaling pathway, upregulating the oncogenic lncRNA SNHG18, which in turn promotes CAR-T cell apoptosis through the miR-211-5p/BRD4 axis (145). These findings suggest that inhibiting SNHG18 expression can enhance the efficacy of CAR-T therapy, offering a novel approach for gastric cancer immunotherapy. CircRNA-based strategies also show considerable therapeutic potential. The ceRNA network orchestrated by circDLG1 increases CXCL12 chemokine expression by sequestering miRNAs, thereby fostering an immunosuppressive niche. Silencing circDLG1 suppresses the proliferation and invasion of gastric cancer cells and reduces resistance to anti-PD-1 therapy, highlighting a multi-ncRNA interplay that could be therapeutically exploited (146).

Chemotherapy and radiotherapy can synergistically enhance ncRNA-mediated antitumor effects through distinct mechanisms. MiR-34a, a tumor suppressor, inhibits the proliferation of gastric cancer cells, and its effect is amplified by oxaliplatin, which induces DNA damage to activate the p53/miR-34a/survivin axis (147). The combination of apatinib and pembrolizumab (Keytruda) modulates the lncRNA CES4/miR-616-5p/DUSP2 axis, inducing apoptosis in gastric cancer cells and providing preclinical evidence for clinical application (148). Radiotherapy sensitization strategies have also been linked to ncRNAs. GAS5, a tumor-suppressive lncRNA, enhances radiosensitivity by promoting DNA damage accumulation via precise regulation of the ATM/p38/MAPK pathway (149). Additionally, circRNA PDSS1 enhances cisplatin sensitivity by sponging miR-515-5p, thereby upregulating integrin alpha-11 (ITGA11) expression (150). Future therapeutic development should prioritize ncRNA-guided adaptive trial designs integrated with artificial intelligence (AI)-driven prediction models to optimize the sequencing of combinatorial treatments. Clinically implementing such rationally engineered regimens has the potential to significantly improve prognosis and increase 5-year survival rates in patients with gastric cancer.

## 6 Research challenges and future prospects

Gastric cancer remains a major global health burden, ranking among the most prevalent malignancies in both incidence and mortality. Innovating therapeutic strategies remains a critical priority in oncology, particularly given the limitations of current treatments. Immune checkpoint blockade (ICB) therapies targeting PD-1/PD-L1 and CTLA-4 have significantly improved outcomes in certain patient subsets. However, the emergence of secondary resistance—largely driven by immunosuppressive reprogramming within the tumor microenvironment mediated by dysfunctional immune cell infiltration—continues to limit clinical response rates. Consequently, optimization of immunotherapeutic approaches remains a key focus of current clinical research. Recent studies have highlighted the pivotal role of ncRNAs in orchestrating the phenotypic remodeling of immune cells, dynamically reshaping the tumor microenvironment, and modulating the expression of immune checkpoints. However, current understanding is limited to a relatively small number of validated ncRNAs, with over 85% of gastric cancer-associated ncRNAs lacking functional characterization in immunological contexts. Bridging the gap between mechanistic insight and clinical application for these uncharacterized ncRNAs will require systematic, multidimensional investigation.

This review synthesizes current knowledge on the regulatory functions of ncRNAs in gastric cancer immunobiology, with particular emphasis on circRNA-miRNA-mRNA networks that modulate immune cell function and checkpoint molecule expression. The discussion also examines the tripartite interactions between ncRNAs, stromal-immune constituents of the tumor immune microenvironment, and cancer cells, revealing novel mechanisms of immune evasion and therapeutic resistance. Despite their therapeutic potential, the clinical translation of ncRNA-based interventions faces three key challenges. Firstly, off-target effects resulting from the lack of tissue-specific expression. Next, drug resistance arising from intratumoral heterogeneity, and then the absence of efficient and safe *in vivo* delivery systems. To address these limitations, an integrated roadmap for next-generation ncRNA therapeutics is proposed: First, multimodal integration of single-cell transcriptomics, high-throughput sequencing, and longitudinal liquid biopsy profiling should be employed to map ncRNA dynamics at high resolution across gastric cancer tumor microenvironment subdomains, enabling the identification of context-dependent regulatory nodes. Second, precision-targeted delivery platforms, such as lipid nanoparticle-based or exosome-based systems, should be engineered to improve tissue specificity and bioavailability. Third, artificial intelligence (AI)-driven predictive models should be developed to forecast therapeutic efficacy and identify synergistic effects between ncRNA-targeted agents and existing immunotherapies. Collectively, these strategies can bridge the gap between ncRNA biology and precision immuno-oncology, offering a blueprint to overcome delivery, specificity, and resistance challenges that currently constrain clinical application in gastric cancer.

NcRNAs have emerged as a pivotal frontier in oncology research, driving advances in cancer immunotherapy. Combinatorial approaches that integrate ncRNA modulation with established immunotherapies, including immune checkpoint inhibitors, adoptive cell therapies, and tumor vaccines, have the potential to significantly improve patient outcomes. Although clinical translation remains challenging, the convergence of multidisciplinary technologies and innovative drug development positions ncRNAs as key molecular tools to overcome the current therapeutic plateau. Future research must strengthen the bidirectional translation between basic science and clinical practice to accelerate the development of personalized immunotherapeutic regimens and ultimately improve long-term survival in patients with gastric cancer.
